# Severe Headache from Carious Teeth

**Published:** 1856-12

**Authors:** J. S. Dixon

**Affiliations:** Near Ashland, Tennessee


### ARTICLE III.

### *Severe Headache from Carious Teeth.*

DRS. LOGAN & WESTMORELAND—When I commenced practice, I, like all other young men, was anxious to get any opportunity to bring myself into notice. This was in the spring of 1852, in Hickman county, Tennessee. It was with me, as with others, all the afflicted were anxious to see the new doctor.

Benjamin Chandler asked me to examine him, rather from curiosity than otherwise, as he had been a standing case for two or three years, and all the medical skill of the country had been exhausted on him, without relief. Age, about forty-five, of temperate habits, was the father of a large family, and had labored on the farm till lately—has suffered for two or three years from a constant, and very severe frontal neuralgia, or headache—has been bled and blistered, purged and puked; besides, the entire routine of tonics, without benefit.

Upon examination, I found his teeth in a very carious condition, but they seldom ached. I immediately concluded that this was the lesion. After some hesitation, he submitted his case to me, exacting the promise, that I should charge him nothing if he received no benefit at my hands. I immediately extracted fifteen teeth and fangs of teeth, and then put him on the use of quinine and tonics, with counter irritation to the forehead and temples. Recovery was slow but sure, and in about twelve months, he was entirely well. I charged him no fee, which was, perhaps, unprofessional; but this put me into business, and after this, I was *the Doctor*.

In cases of hemicrania and frontal neuralgia, I invariably examine the teeth, and if I find them carious, I advise extraction, and this seldom fails to cure; and I have had several of my own teeth extracted on this account, followed by entire relief.

You shall know the sequel of my case of abnormal menstruation. Yours, truly,

J. S. DIXON.

*Near Ashland, Tennessee.*

